# Intraoperative MEP and EMG during the wake-up phase as adjuncts to the wake-up test in severe spinal deformity surgery

**DOI:** 10.3389/fsurg.2026.1739334

**Published:** 2026-06-18

**Authors:** Birong Gao, Yaolong Deng, Jian Chen, Juncheng Li, Jingfan Yang, Junlin Yang, Yaqing Zhang

**Affiliations:** 1 Department of Pediatric Operating Room, Xinhua Hospital Affiliated to Shanghai Jiao Tong University School of Medicine, Shanghai, China; 2 Spine Center, Xinhua Hospital Affiliated to Shanghai Jiao Tong University School of Medicine, Shanghai, China; 3 Pediatric Orthopaedics, Xinhua Hospital Affiliated to Shanghai Jiao Tong University School of Medicine, Shanghai, China

**Keywords:** electromyography, motor evoked potentials, neurological prognosis, severe spinal deformity surgery, wake-up test

## Abstract

**Background:**

This study aimed to evaluate the adjunctive value of intraoperative motor evoked potential (MEP) recovery and electromyographic (EMG) activity during the wake-up phase in predicting postoperative neurological outcomes in high-risk patients with intraoperative MEP alerts during severe spinal deformity surgery.

**Methods:**

A retrospective analysis was conducted in 111 patients with intraoperative MEP alerts who underwent thoracic osteotomy for severe spinal deformity between 2008 and 2022. Postoperative neurological outcomes were compared according to wake-up test responses, MEP recovery, and EMG activity during the wake-up phase. The predictive performance of each modality and their combinations for immediate postoperative complete lower-limb paralysis was assessed. The time required to obtain interpretable intraoperative responses during the wake-up phase was also analyzed.

**Results:**

Among 59 patients without immediate postoperative complete lower-limb paralysis, 46 (78.0%) recovered motor function immediately after surgery, and all recovered within 1 month. Among 52 patients with immediate postoperative complete lower-limb paralysis, 18 (34.6%) recovered within 1 month, 47 (90.4%) recovered within 1 year, and 5 (9.6%) failed to return to baseline neurological status by final follow-up. The false-positive rate for predicting complete paralysis was 20.3% for the wake-up test alone, 10.2% for MEP recovery alone, and 33.9% for EMG alone. This rate decreased to 6.8% when MEP and EMG were combined, and to 0% when wake-up test, MEP, and EMG findings were considered together. In patients without complete paralysis, MEP and EMG responses were obtained earlier during arousal than direct wake-up test responses.

**Conclusions:**

In high-risk severe spinal deformity cases with intraoperative MEP alerts, MEP recovery and EMG activity during the wake-up phase may provide useful complementary information to the traditional wake-up test for assessing postoperative neurological risk. A multimodal intraoperative assessment strategy may improve the interpretation of neurological status during arousal and help reduce false-positive findings in selected patients.

## Introduction

Spinal cord injury is one of the serious complications during spinal deformity correction, especially in the severe rigid spinal deformity surgery, which may need high grade osteotomy and large correction (1). Nonetheless, complex screw placement, osteotomy, and correction maneuvers have markedly increased the risk of spinal cord ischemia injury, leading to postoperative neurological deficit (2). If not treated in a timely manner, it can lead to reduced thoracic volume, impaired mobility, and a reported incidence of neurological complications ranging from 4.0% to 25.7%, with the risk of permanent paralysis reaching as high as 6.3% (3, 4).

The Intraoperative spinal cord electrophysiological monitoring is essential to prevent these severe complications. Intraoperative spinal cord monitoring—including motor evoked potentials (MEP), somatosensory evoked potentials, descending neurogenic evoked potentials, free-run and triggered electromyography (EMG)—has been proven effective and is widely used in clinical practice (5, 6). However, various factors may influence the neuromonitoring accuracy which necessitate the wake-up test to prevent neurological complication (7). The wake-up test is applied during the surgery when neuromonitoring changes occur to indicate the spinal cord injury (8). The wake-up test is used for early monitoring and potentially prevention of the neurological complication and was appied firstly by Vauzella and Stagmara in 1973 (9).

Despite its historical value, the wake-up test presents several significant limitations. It interrupts the surgical workflow, depends heavily on patient cooperation, and may cause distress or recall in patients, particularly if analgesia or sedation is inadequate (10). Additionally, the results of the wake-up test are binary and subjective, lacking the precision and continuity provided by electrophysiological monitoring. Furthermore, the test may be confounded by residual neuromuscular blockade or delayed arousal, potentially leading to false-positive or false-negative results. Notably, clinical observations consistently show that as long as voluntary movement of the lower limbs is present during the wake-up test—even if minimal, such as a single toe movement—postoperative neurological recovery is generally favorable, even in patients who ultimately exhibit objective evidence of spinal cord injury. This highlights the clinical importance of correctly identifying a reassuring wake-up response, as even minimal voluntary lower-limb movement may predict favorable neurological recovery.

To address these challenges, our team explored whether intraoperative MEP recovery and free-run EMG activity during the wake-up phase could provide objective and continuous adjunctive information to complement the traditional wake-up test. This study retrospectively reviewed data from 111 patients who experienced intraoperative MEP-positive events and wake-up tests during severe thoracic osteotomy correction surgery. By analyzing MEP recovery, EMG activity, and wake-up test responses during the wake-up phase, and comparing these findings with immediate postoperative neurological outcomes, this study aimed to evaluate their complementary value in predicting early postoperative neurological status in high-risk severe spinal deformity patients with intraoperative MEP alerts. The findings of this study should be interpreted within the specific context of high-risk deformity correction cases with intraoperative MEP alerts, rather than routine deformity surgery or all neuromonitoring scenarios.

## Materials and methods

### Study design and patient selection

This retrospective single-center study reviewed 536 consecutive patients who underwent corrective osteotomy for severe thoracic spinal deformity at our institution between May 2008 and April 2022. Among them, 111 patients experienced a significant intraoperative motor evoked potential (MEP) alert and subsequently underwent an intraoperative wake-up test, and these patients constituted the study cohort. The inclusion criteria were as follows: patients had a primary thoracic deformity with a coronal and/or sagittal Cobb angle of at least 90°, underwent three-column osteotomy (Schwab grade ≥3), received multimodal intraoperative neuromonitoring with successful baseline acquisition including MEP monitoring, developed a significant intraoperative MEP alert with complete intraoperative monitoring records available, underwent a wake-up test with corresponding electrophysiological recordings during the wake-up phase, and had postoperative neurological follow-up for at least 1 year. Because of this design, the present study specifically focused on a high-risk subgroup of patients with intraoperative MEP alerts during severe spinal deformity correction, rather than on routine deformity surgery or all neuromonitoring scenarios.

### Clinical data collection

Demographic and preoperative variables included sex, age at surgery, primary coronal Cobb angle, and primary sagittal kyphotic Cobb angle. Intraoperative variables included the occurrence of MEP alerts, changes in MEP signals after intervention, wake-up test responses, free-run electromyography (EMG) activity during the wake-up phase, and MEP recovery during the wake-up phase. Postoperative neurological status was assessed using lower-extremity muscle strength graded by the Medical Research Council (MRC) scale immediately after surgery and during follow-up. In addition, the time required to obtain interpretable responses from the wake-up test, EMG activity, and MEP recovery during arousal was recorded. For the purpose of evaluating predictive performance, the primary clinical reference outcome was immediate postoperative motor status, categorized as complete vs. non-complete lower-limb paralysis.

### Anesthesia management

To minimize interference from inhalational anesthetics on intraoperative neuromonitoring, particularly MEP and EMG, total intravenous anesthesia was used in all cases. Anesthesia was induced with propofol (1.5–2.0 mg/kg), fentanyl (3–5 µg/kg), and a muscle relaxant, usually cisatracurium (0.15–0.2 mg/kg), to facilitate endotracheal intubation. Maintenance was achieved with remifentanil (0.2–0.5 µg/kg/min) and propofol (5–6 mg/kg/h). After induction, muscle relaxants were avoided or used only in minimal doses in order to reduce suppression of MEP signals and to minimize interference with wake-up assessment. When a wake-up test was required, anesthetic agents were discontinued in advance according to the intraoperative protocol to allow patient arousal and facilitate neurological evaluation. Because wake-up responses, MEP recovery, and EMG activity may all be influenced by anesthetic depth, residual neuromuscular blockade, blood pressure, temperature, and other physiological variables, these factors were routinely evaluated during intraoperative troubleshooting.

### Intraoperative electrophysiological monitoring and alert criteria

Intraoperative neuromonitoring was performed using one of the following systems: Nicolet Endeavor CR 16, Xltek Protektor 32, Cadwell Cascade Pro 32, or Medtronic NIM Eclipse 32. MEP and free-run EMG were monitored continuously in an alternating real-time sequence throughout the procedure. For MEP monitoring, an all-or-none principle was used as the warning criterion. A significant MEP alert was defined as marked loss or disappearance of previously established MEP responses after exclusion of systemic, anesthetic, and technical causes. For free-run EMG, activity patterns were classified as burst or train discharges. Burst activity was defined as a single nonrepetitive discharge, whereas train activity was defined as a prolonged repetitive neurotonic discharge. Either pattern was recorded as evidence of neural irritation or activation during the wake-up phase. All neuromonitoring alerts were required to show a clear temporal and logical relationship with high-risk surgical maneuvers, including pedicle screw placement, osteotomy, and deformity correction. Before confirming a true alert, technical malfunction, anesthetic effects, neuromuscular blockade, hemodynamic instability, hypothermia, and other physiological confounders were systematically excluded.

### Standardized intervention protocol after intraoperative MEP alert

When a significant intraoperative MEP alert occurred, a standardized stepwise protocol was initiated. First, systemic and technical causes were assessed, including device function, electrode integrity, neuromuscular blockade, anesthetic depth, blood pressure, body temperature, and other relevant physiological parameters. If no reversible systemic cause was identified, high-risk surgical maneuvers were immediately suspended, blood pressure was increased, and anesthesia was lightened. If the alert persisted, corticosteroids were administered prophylactically, and the spinal cord tension and osteotomy site were explored with decompression as needed. If MEP signals still failed to recover adequately, a wake-up test was performed. If correction had already been applied, partial release of correction was attempted to restore the spine toward its pre-correction state. If signal loss persisted, additional decompression and, when necessary, implant removal while maintaining spinal stability were considered until recovery occurred. If MEP signals failed to recover despite these measures, the procedure was terminated.

### Definition of wake-up phase findings and outcome grouping

If MEP signals did not recover adequately after intervention, a wake-up test was performed to assess intraoperative motor function. During the wake-up phase, anesthetic depth was reduced and sedative agents were withheld according to protocol. To improve clarity and avoid ambiguity, wake-up phase findings were defined in descriptive terms rather than as positive or negative events. A wake-up test response was considered present when voluntary lower-limb movement was observed, even if limited to minimal distal movement such as toe motion, and absent when no voluntary lower-limb movement could be elicited. EMG activity during the wake-up phase was considered present when clear bilateral lower-limb free-run EMG activity was detected and absent when no significant lower-limb EMG activity was recorded. MEP recovery during the wake-up phase was considered present when substantial recovery of MEP signals was observed during a recording obtained under stable airway and physiological conditions, and absent when no substantial recovery occurred.

The predictive value of each monitoring modality was analyzed separately and in combination, including wake-up test response alone, MEP recovery alone, EMG activity alone, combined MEP recovery and EMG activity, and the combination of wake-up test response, MEP recovery, and EMG activity. In the combined models, the presence of any reassuring sign, including voluntary movement, MEP recovery, or detectable bilateral EMG activity, was interpreted as a favorable intraoperative finding. Immediate postoperative neurological outcomes were dichotomized into complete lower-limb paralysis and non-complete lower-limb paralysis. This binary outcome served as the main clinical reference outcome for evaluating the predictive performance of intraoperative wake-up phase findings. All patients undergoing wake-up testing or wake-up-phase MEP assessment received standardized preoperative instruction to improve intraoperative cooperation.

### Statistical analysis

Demographic and clinical data were summarized using descriptive statistics. Continuous variables were expressed as mean ± standard deviation and compared using the independent-samples t test. Categorical variables were compared using the chi-square test. The predictive performance of wake-up test response, MEP recovery, EMG activity, and their combinations for immediate postoperative complete lower-limb paralysis was evaluated primarily by comparing false-positive findings across different monitoring strategies. The time required to obtain interpretable intraoperative responses during the wake-up phase was also compared among modalities. Statistical analyses were performed using SPSS version 19.0 (SPSS Inc., Chicago, IL, USA), and a two-sided *P* value < 0.05 was considered statistically significant.

## Results

### Patient characteristics

A total of 111 patients were included in this study, comprising 38 males (34.2%) and 73 females (65.8%), with a mean age of 27.0 ± 10.8 years (range, 9–66 years). The underlying diagnoses included idiopathic scoliosis in 38 patients (34.2%), congenital scoliosis in 54 (48.7%), neurofibromatosis-associated scoliosis in 6 (5.4%), neuromuscular scoliosis in 6 (5.4%), tuberculosis-associated scoliosis in 1 (0.9%), Marfan syndrome in 2 (1.8%), and revision surgery for congenital scoliosis in 4 (3.6%). The mean preoperative major coronal Cobb angle was 123.03 ± 25.24° (range, 60°–210°), and the mean major kyphotic Cobb angle was 114.95 ± 25.30° (range, 40°–180°). The osteotomy procedures included pedicle subtraction osteotomy (PSO) in 28 patients (25%), posterior vertebral column decancellation (PVCD/PD) in 13 (12%), vertebral column resection (VCR) in 60 (54%), and vertebral column resection with satellite techniques (VCRs) in 10 (9%). The mean operative duration was 465.92 ± 122.92 min (Table 1).

**Table 1:** Baseline demographic, radiographic, and operative characteristics of the 111 high-risk patients with intraoperative MEP alerts.

Characteristic	Value
Gender(Male/Female)	38/73
Age	27 ± 10.8 (9–66)
Diagnosis	
Idiopathic scoliosis	38
Congenital scoliosis	54
Neurofibromatosis-associated scoliosis	6
Neuromuscular scoliosis	6
Tuberculosis-related scoliosis	1
Marfan syndrome	2
Revision surgeries	4
Cobb angle of major scoliotic curve	(123.03 ± 25.24)°(60°–210°)
Cobb angle of major kyphotic curve	(114.95 ± 25.30)°(40°–180°)
Type of osteotomy	
PSO	28
PD	13
VCR	60
VCRs	10
Surgical time	465.92 ± 122.92

### Postoperative neurological recovery

Of the 111 patients, 59 did not develop immediate postoperative complete lower-limb paralysis and were therefore classified into the non-complete paralysis group. Among these patients, 46 (78.0%) had already returned to their preoperative neurological status at the initial postoperative assessment, and the remaining 13 achieved complete recovery within 1 month after surgery. In contrast, 52 patients developed immediate postoperative complete lower-limb paralysis. In this group, 18 patients (34.6%) recovered to baseline neurological function within 1 month, and 47 (90.4%) recovered within 1 year. However, 5 patients (9.6%) failed to return to their baseline neurological status by the final follow-up. These findings indicate that patients without immediate postoperative complete paralysis generally had a favorable early neurological course, whereas complete paralysis immediately after surgery was associated with delayed recovery and a higher risk of persistent deficit (Table 2).

**Table 2:** The follow-up of neurological function data for Two postoperative paralysis groups.

Follow-up time point	Non-complete paralysis group (*n* = 59)	Complete paralysis group (*n* = 52)
Postoperative (-) Immediately	46/59	0/52
Postoperative (-) 1M	59/59	18/52
Postoperative (-) 1Y	59/59	47/52

Complete lower-limb paralysis was defined as absence of voluntary lower-extremity motor function immediately after surgery; non-complete paralysis included preserved or partially preserved motor function.

### Predictive performance of wake-up test, MEP recovery, and EMG activity during the wake-up phase

We then compared the predictive performance of wake-up test response, MEP recovery, and free-run EMG activity during the wake-up phase for identifying immediate postoperative complete lower-limb paralysis. When considered individually, the false-positive rate was 20.3% for the wake-up test response, 10.2% for MEP recovery during the wake-up phase, and 33.9% for EMG activity alone. No statistically significant difference was found among these individual modalities (all *P* > 0.05).

When MEP recovery and EMG activity were evaluated together during the wake-up phase, the false-positive rate decreased to 6.8% (Table 3). Although this reduction did not reach conventional statistical significance compared with the wake-up test alone (*P* = 0.06), it suggested improved discrimination when these two electrophysiological findings were interpreted in combination. Most notably, when wake-up test response, MEP recovery, and EMG activity were considered together, the false-positive rate for predicting immediate postoperative complete lower-limb paralysis decreased to 0%, which was significantly lower than that of the wake-up test alone (*P* < 0.05).

**Table 3:** Comparison of accuracy in wake-up procedure outcome indicators.

Outcome group/metric	Wake-up test		Wake-up MEP		Wake-up EMG		Wake-up EMG + MEP		Wake-up + Wake-up EMG + MEP	
	(−)	(+)	(−)	(+)	(−)	(+)	(−)	(+)	(−)	(+)
Non-complete paralysis group	47	12	53	6	39	20	55	4	59	0
Complete paralysis group	0	52	0	52	0	52	0	52	0	52
False positive rate	20.3%		10.2%		33.9%		6.8%		0	
P	-		0.18		0.13		0.06		<0.05	
False negative rate	0		0		0		0		0	

Wake-up test response indicates voluntary lower-limb movement present during the wake-up phase. MEP recovery indicates substantial recovery of motor evoked potentials during the wake-up phase. EMG activity indicates detectable bilateral lower-limb free-run electromyographic activity during arousal. In the combined models, the presence of any reassuring sign was interpreted as a favorable intraoperative finding. MEP, motor evoked potential; EMG, electromyography.

In addition, among patients without complete paralysis, interpretable MEP recovery was obtained at a mean time of 18.6 ± 15.6 min during arousal, and EMG activity was detected at 24.3 ± 11.5 min, whereas interpretable wake-up test responses were obtained at 25.9 ± 11.5 min. These findings suggest that electrophysiological signals, particularly MEP recovery, may provide earlier adjunctive information than voluntary movement alone during the wake-up phase.

## Discussion

Although the effectiveness of intraoperative spinal cord monitoring has been confirmed and widely applied, the wake-up test is still considered an important reference method for intraoperative judgment of motor pathway injury in selected situations (11). Performing a wake-up test during surgery can provide direct clinical information regarding motor function when neuromonitoring changes occur. However, in clinical practice, this method presents several important limitations (12). Firstly, it only assesses gross motor function and provides no information about sensory pathways or individual nerve roots (13). Secondly, it is unsuitable for continuous or repeated monitoring during surgery due to the interruption of anesthesia and the time required for arousal. Additionally, it is difficult to perform in infants, cognitively impaired individuals, or those with communication difficulties (14). Complications such as endotracheal tube displacement, hemorrhage, and patient agitation may occur during the wake-up process (13). Moreover, factors like prolonged surgical duration, delayed anesthetic metabolism, and limited intraoperative anesthetic management experience may prolong the wake-up process, unnecessarily extend surgical time, and delay timely intervention, potentially increasing the risk of neural injury and poor recovery outcomes (8, 15). False positives may also arise due to residual sedation or patient-specific factors, leading to misinterpretation of neurological function (16–18). In addition, wake-up responses, MEP recovery, and EMG activity during arousal may all be influenced by anesthetic depth, residual neuromuscular blockade, blood pressure, temperature, and other physiological variables. Therefore, there is a clear need to explore objective adjunctive intraoperative strategies to accurately evaluate spinal cord integrity—especially in high-risk severe spinal deformity surgery, where timely and reliable intraoperative assessment is critical.

Our findings emphasize the clinical importance of distinguishing between complete and incomplete spinal cord injuries during surgery. The data reveal that patients without complete lower limb paralysis in the immediate postoperative period generally have a favorable prognosis, with nearly all returning to baseline function within a month. In contrast, those with complete paralysis face a significantly higher risk of prolonged or permanent neurological deficits. These observations reinforce the utility of MEP and EMG monitoring during the wake-up phase, particularly as complementary tools for interpreting intraoperative neurological status in patients with persistent MEP alerts. The ability to identify patients with potentially reversible injuries intraoperatively may allow for timely surgical adjustments and postoperative interventions that enhance long-term outcome. Therefore, based on these findings, it is crucial to accurately and promptly identify motor responses during the wake-up test while taking potential confounding factors into account. One of the most critical limitations of the wake-up test is its potential for false-positive results10, 15. These occur when the patient appears unresponsive or shows abnormal movement during the test, suggesting possible neurological injury, when in fact no real damage exists. Such misleading outcomes are often caused by residual effects of anesthetic agents, incomplete reversal of neuromuscular blockade, or variability in patient arousal thresholds (19). In these cases, the surgical team may halt or reverse corrective procedures unnecessarily, leading to suboptimal outcomes or prolonged operative time. Moreover, the subjective nature of interpreting patient movement—and the lack of real-time, quantitative data—makes the wake-up test especially vulnerable to misjudgment. This unreliability underscores the need for more objective and physiologically grounded adjunctive monitoring strategies to complement traditional wake-up assessment. Importantly, we observed that in many cases where voluntary lower limb movement was present during the wake-up test—even in the presence of abnormal intraoperative neurophysiologic signals—patients ultimately exhibited favorable postoperative neurological outcomes. This suggests that when a reassuring wake-up response is present (i.e., the patient is able to move the lower limbs), the likelihood of severe motor pathway injury is low, and the finding may be considered clinically reassuring. Accurately distinguishing between a true-positive and a false-positive wake-up test result is therefore essential. If a reassuring wake-up response can be confidently confirmed, surgical procedures may be resumed without delay or unnecessary intervention, reducing operative time, minimizing anesthetic exposure, and potentially preventing secondary complications. Thus, one of the critical values of our study lies in refining the intraoperative interpretation of wake-up test outcomes, especially in recognizing and eliminating false-positive scenarios, which in turn improves surgical efficiency and patient safety.

In this study, we compared traditional wake-up test results with intraoperative MEP recovery and EMG activity observed during the wake-up process to assess their predictive value for postoperative complete lower limb paralysis. Our findings showed that when reassuring electrophysiological findings were present during the wake-up phase, particularly when MEP recovery and EMG activity were interpreted together, none of the patients developed complete postoperative lower-limb paralysis. In contrast, the traditional wake-up test alone showed a false-positive rate of 20.3% when predicting complete paralysis. When evaluated individually, MEP recovery during the wake-up process showed a false-positive rate of 10.2%, while EMG monitoring during the same period showed a false-positive rate of 33.9%. Although neither showed statistically significant differences compared to the traditional wake-up test (*P* > 0.05), combining MEP recovery and EMG activity during the wake-up phase reduced the false-positive rate to 6.8%, although this difference represented only a trend and did not reach conventional statistical significance (*P* = 0.06). Most notably, when the traditional wake-up test was combined with intraoperative MEP recovery and EMG findings, the false-positive rate for predicting complete lower limb paralysis dropped to 0%. This combined approach showed a statistically significant improvement compared to the traditional wake-up test alone (*P* < 0.05). These results underscore the potential of integrating real-time electrophysiological data into the intraoperative wake-up assessment, not only to enhance diagnostic accuracy but also to reduce unnecessary surgical interruptions due to false alarms.

These findings highlight several important considerations for optimizing intraoperative neurological monitoring in severe spinal deformity surgery. First, in this specific high-risk cohort with intraoperative MEP alerts, combining wake-up-phase MEP recovery and EMG activity provided more reassuring information than either modality alone. Rather than suggesting replacement of the wake-up test, our results support the use of these modalities as complementary indicators during intraoperative decision-making. This is particularly relevant in clinical scenarios where delayed arousal, residual neuromuscular blockade, or suboptimal patient response may complicate the interpretation of the wake-up test. From a surgical workflow perspective, the reduced false-positive rate has practical implications. Fewer unnecessary interruptions in deformity correction may be required due to misinterpreted wake-up results, leading to more efficient intraoperative management and more stable intraoperative conditions. Additionally, when electrophysiological signals provide information consistent with preserved neurological function, reliance on the wake-up test—which is inherently subjective and disruptive—may be reduced in selected circumstances, rather than eliminated. This may be particularly beneficial in pediatric patients or others unable to cooperate effectively with traditional wake-up assessments. Moreover, our study supports the development of a more nuanced intraoperative neurological monitoring strategy, wherein wake-up test results are no longer interpreted in isolation, but rather in the context of concurrent neurophysiological signals. This integrative approach may be especially valuable in high-risk cases, such as those requiring multiple-level osteotomies or aggressive deformity correction. Our findings demonstrate that in patients with incomplete paralysis, neurophysiological monitoring modalities—specifically MEP (18.6 ± 15.6 min) and EMG (24.3 ± 11.5 min)—provided interpretable findings earlier than the traditional wake-up test (25.9 ± 11.5 min) during intraoperative arousal. This temporal advantage suggests that multimodal intraoperative monitoring may provide earlier adjunctive information during emerging neural compromise, potentially allowing for faster intervention compared to reliance on wake-up testing alone.

Several limitations of this study should be acknowledged. First, this was a retrospective single-center study, which may limit the generalizability of the findings. Second, the study population was restricted to patients with severe spinal deformity and intraoperative MEP alerts who underwent wake-up testing, and therefore the conclusions should not be extrapolated to routine deformity surgery or all neuromonitoring scenarios. Third, although we compared false-positive findings among different assessment strategies, a more comprehensive diagnostic accuracy analysis, including sensitivity and specificity, would further strengthen the conclusions. Finally, despite the use of a standardized anesthetic and troubleshooting protocol, wake-up responses, MEP recovery, and EMG activity may still have been affected by residual anesthetic and physiological confounding factors. In addition, the absence of multivariable modeling limited our ability to assess the independent contribution of each intraoperative finding to postoperative neurological risk.

In summary, while the wake-up test remains an important tool for intraoperative assessment of spinal cord function, its limitations underscore the need for objective adjunctive methods. Our findings support the clinical utility of combining intraoperative MEP and EMG monitoring during the wake-up process to improve the interpretation of neurological risk, reduce false-positive results, and enhance intraoperative decision-making in high-risk patients with intraoperative MEP alerts. Future prospective studies with larger sample sizes and multicenter collaboration are warranted to further validate these findings and potentially establish new intraoperative neuromonitoring protocols.

## Conclusion

Intraoperative assessment of motor evoked potential (MEP) recovery and electromyography (EMG) activity during the wake-up phase may provide objective complementary information for evaluating spinal cord function in high-risk patients undergoing corrective surgery for severe thoracic spinal deformity. Our findings suggest that the combined interpretation of MEP and EMG during the wake-up phase may help reduce false-positive findings associated with the traditional wake-up test and may assist intraoperative decision-making. Furthermore, integrating wake-up test response with concurrent MEP and EMG findings may improve the assessment of postoperative neurological risk in selected patients with intraoperative MEP alerts.

## Data Availability

The original contributions presented in the study are included in the article/Supplementary Material, further inquiries can be directed to the corresponding authors.
